# Elevated pleural effusion IL-17 is a diagnostic marker and outcome predictor in lung cancer patients

**DOI:** 10.1186/2047-783X-19-23

**Published:** 2014-05-08

**Authors:** ChunHua Xu, LiKe Yu, Ping Zhan, Yu Zhang

**Affiliations:** 1 First Department of Respiratory Medicine, Nanjing Chest Hospital, 215 Guangzhou Road, Nanjing 210029, China

## Abstract

**Background:**

Interleukin 17 (IL-17) is a proinflammatory cytokine produced mainly by CD4^+^ T-lymphocytes and may be important in tumor cell growth and progression. In this study, we aimed to evaluate the diagnostic and prognostic value of pleural effusion levels of IL-17 in lung cancer patients with malignant pleural effusion (MPE).

**Methods:**

Pleural effusion samples were collected from 78 lung cancer patients with MPE and from 45 patients with nonmalignant pleural effusion. Pleural fluid concentrations of IL-17 were measured by using enzyme-linked immunosorbent assays.

**Results:**

Malignant effusion exhibited higher IL-17 levels than nonmalignant effusion (20.49 ± 5.27 pg/ml vs. 13.16 ± 2.25 pg/ml; *P* < 0.01). Lung cancer patients with pleural fluid IL-17 levels below 15 pg/ml had longer overall survival than those patients with higher levels (10.8 months vs. 4.7 months; *P* < 0.05). On the basis of multivariate analysis, we found that pleural fluid IL-17 level was an independent prognostic factor in lung cancer patients with MPE.

**Conclusions:**

Measurement of IL-17 levels might be a useful diagnostic and prognostic test for lung cancer patients with MPE.

## Background

Lung cancer is the leading cause of cancer-related mortality in the world [1]. Approximately 15% of lung cancer patients have pleural effusion at the time of initial diagnosis, and 50% develop it later in the course of their disease [2]. Differentiating malignant from nonmalignant pleural effusion is a critical problem, and conventional methods have proven inadequate [3-6]. Pleural fluid cytology has traditionally been the analytical method of choice for the detection of tumor cells in pleural fluid. However, sensitivity varies between 30% and 60% [7], and blindly obtained pleural needle biopsy specimens offer little additional sensitivity [8]. Although the presence of tumor cells in pleural effusion is a diagnostic marker of malignant pleural effusion (MPE), the probability of finding them is low. For negative cytology pleural effusion, some of the currently used indices, such as carcinoembryonic antigen, neuron-specific enolase and cytokeratin 19 fragments (CYFRA 21-1), have a certain extent of differential value; however, their specificity and sensitivity are limited [9,10]. Therefore, searching for new indices is very important.

In addition to diagnostic issues, patients with MPE have a poor prognosis and are difficult to treat effectively [11]. Despite advances in treatment modalities, the overall survival (OS) is still very short. The present standard treatment is to evacuate the pleural fluid, followed by intravenous chemotherapy or intrapleural chemotherapy [12]. However, it was found that not all patients were benefited from the addition of chemotherapy, especially in patients with short OS. Therefore, prognostic assessment of the patient is essential. Hsu *et al*. proved that the expression level of angiogenetic biomarkers was significantly correlated with patient survival and pleural effusion control [13]. In addition, researchers in recent molecular and genetic profiling studies identified several markers as diagnostic and prognostic factors of lung cancer.

The improved understanding of pleural effusion immunopathogenesis could lead to the development of immunodiagnostic tools to facilitate its differential diagnosis. Investigators in large-scale studies have reported that lymphocytes play an important role in the pathogenesis of pleural effusion [14-17]. CD4^+^ T cells can be differentiated into interleukin 17 (IL-17)-producing T helper (Th17) cells. Th17 cells produce unique cytokines, including IL-17A, IL-17F and IL-22. These cytokines play a role in inflammation and cancers [18-21]. In previous studies, researchers have reported that the levels of IL-17 are elevated in pleural effusion [22,23]. However, whether there is a significant difference in their expression level as well as in the relationship between pleural fluid IL-17 concentrations and the prognosis for patients with lung cancer have not been evaluated to date.

In our present study, we assessed pleural effusion of various etiologies in 123 patients to determine whether IL-17 pleural fluid could be used as a diagnostic indicator of lung cancer and a predictor of survival time. We found that IL-17 is elevated in malignant effusion caused by lung cancer, as well as being potentially predictive of survival outcome.

## Methods

### Patients

The study included 123 consecutive patients with pleural effusion who were recruited from the Nanjing Chest Hospital from January 2009 to December 2010. All cases of pleural effusion had a definite etiology documented by examination of effusion biochemistry, cytology, pleural biopsy, percutaneous biopsy, endoscopic examination and clinical follow-up. The characteristics of the patients are summarized in Table 1. The patient group included 70 men and 53 women with a median age of 65 years. Of the total sample, 78 patients (63.4%) had MPE and 45 (36.6%) had nonmalignant pleural effusion. The OS time in patients with MPE was measured from the time of diagnosis to the date of death or the last follow-up.

**Table 1:** **Patient characteristics**^**a**^

**Variables**	**MPE**	**BPE**	***P*-value**
Patients, *n*	78	45	
Mean age (±SD), years	56.3 ± 12.5	55.6 ± 11.7	>0.05
Male/female	36/42	34/11	>0.05
MPE			
Adenocarcinoma	67	ND	
Squamous cell carcinoma	5	ND	
Small-cell lung carcinoma	6	ND	
BPE			
Tuberculous	ND	28	
Parapneumonic	ND	12	
Heart failure	ND	5	
Diagnostic method			
Biochemistry	ND	30	
Cytology	50	ND	
Pleural biopsy	6	8	
Percutaneous biopsy	12	4	
Endoscopic examination	10	3	

^a^BPE, Benign pleural effusion; MPE, Malignant pleural effusion; ND, No data.

This study was approved by the Ethics Committee of Nanjing Chest Hospital, and informed consent was obtained from each patient.

### Diagnostic criteria for pleural effusion

The diagnostic criteria for MPE were as follows. Cytological evidence of malignant cells present in pleural effusion or in biopsy specimens. Tuberculous (TB) pleural effusion was diagnosed according to the following findings: identification of acid-fast bacilli in pleural fluid, caseous granulomas in a pleural biopsy specimen and a high level of pleural fluid adenosine deaminase (>40 U/L) with an improvement of the pleurisy after anti-TB therapy. Parapneumonic effusion was characterized by any pleural effusion associated with pneumonia and response to antibiotics. Patients with pleural empyema were also included in this group. Effusions related to heart failure were collected from patients with documented heart failure but without neoplastic or other disease.

### Sample collection and determination of IL-17 concentration

Fresh pleural effusion specimens were collected before treatment and centrifuged at 1,500 × *g* for 10 minutes at -4°C. The supernatants were immediately stored at -80°C until use. The IL-17 concentrations were determined by enzyme-linked immunosorbent assay (ELISA) with the commercial human IL-17 Ready-SET-Go! ELISA Kit (eBioscience, San Diego, CA, USA). All assays were run in duplicate, with dilutions as appropriate, and the technicians were blinded to clinical data.

### Statistical analyses

All statistical analyses of differences between malignant effusions and nonmalignant pleural effusion were performed using the Mann–Whitney *U* test. The diagnostic accuracy of IL-17 in discriminating between lung cancer with malignant and nonmalignant pleural effusion was compared by constructing receiver operating characteristic (ROC) curves. The optimum cutoff point from the ROC analysis was established by selecting the value that provided the greatest sum of sensitivity and specificity. Survival analyses were performed using the Kaplan–Meier method, and significant differences in survival rates were compared using the logrank test. The Cox proportional hazards regression model was used to compare the relative influence of different prognostic factors. *P* < 0.05 was considered to indicate statistical significance.

## Results

### Levels of IL-17 in pleural effusion

**Figure 1:**
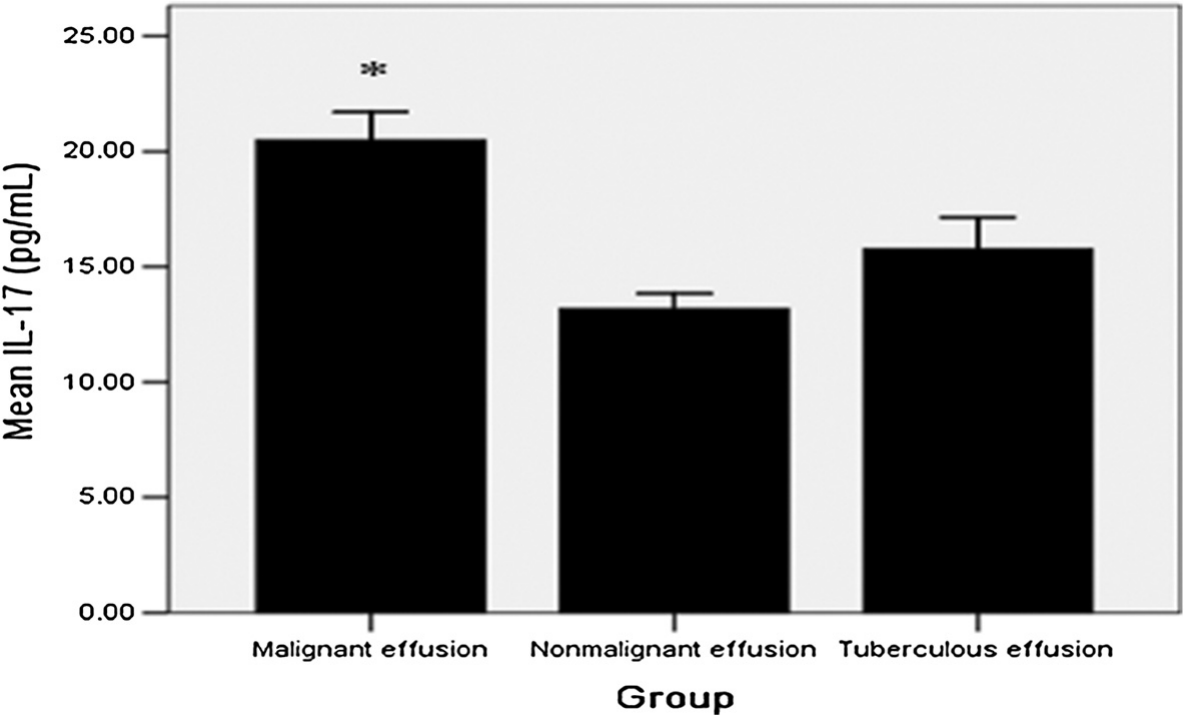
**Levels of interleukin 17 in pleural effusion.** Malignant effusion exhibited higher interleukin 17 (IL-17) concentrations than in nonmalignant effusion and tuberculous effusion. *compared with nonmalignant effusion group and TB effusion group, *P* < 0.05 for both.

As shown in Figure 1, patients with malignant effusion exhibited higher IL-17 concentration than those with nonmalignant pleural effusion (20.49 ± 5.27 pg/ml vs. 13.16 ± 2.25 pg/ml; *P* = 0.004). Pleural fluid IL-17 concentrations in patients with malignant effusion were higher than in patients with TB effusion (20.49 ± 5.27 pg/ml vs. 17.43 ± 5.39 pg/ml; *P* = 0.021).

### Diagnostic value of IL-17 in malignant pleural effusion

**Figure 2:**
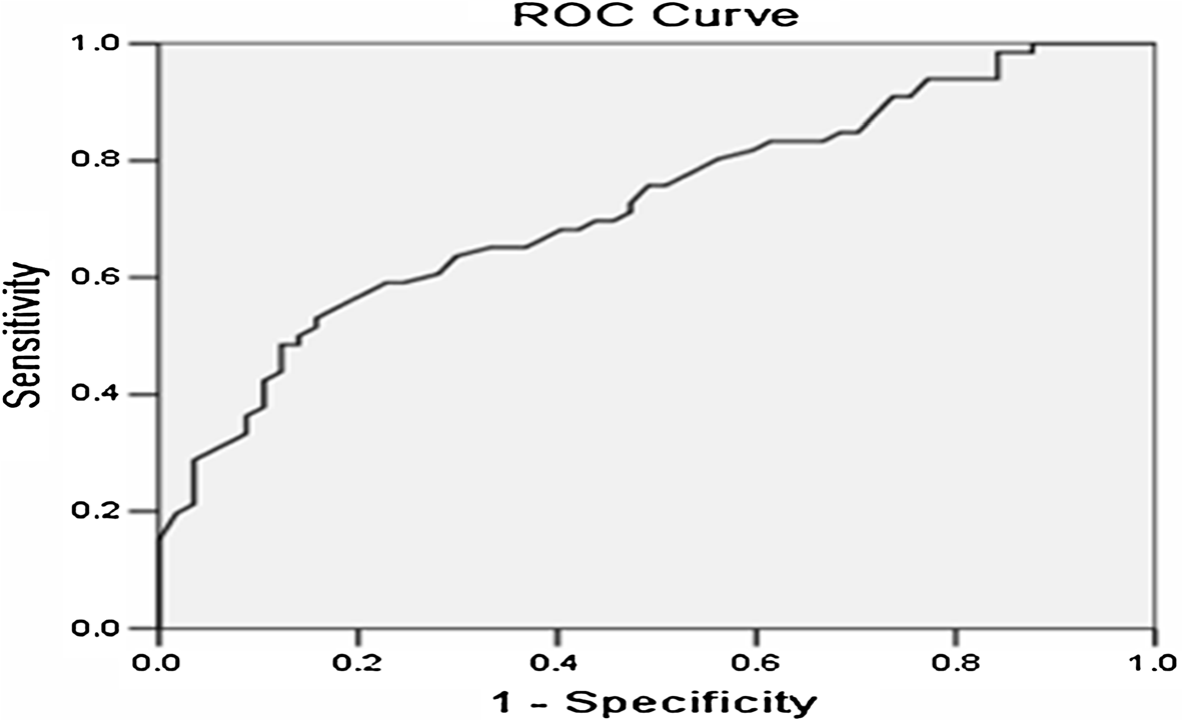
**Receiver operating characteristic curve of interleukin 17 for the differential diagnosis of malignant and nonmalignant effusion.** ROC, Receiver operating characteristic.

ROC curve analysis was carried out to assess the IL-17 concentrations in patients with MPE. The area under the ROC curve was 0.724 (95% confidence interval = 0.635 to 0.812). The best efficacy was observed at 15 pg/ml. Using a cutoff value of 15 pg/ml, IL-17 had a sensitivity of 79.5% (62 of 78 patients), specificity of 91.1% (41 of 45 patients), accuracy of 83.7% (103 of 123 patients), positive predictive value of 93.9% (62 of 66 patients) and negative predictive value of 71.9% (41 of 57 patients) (Figure 2).

### Relationship between IL-17 concentration and clinicopathological factors in lung cancer patients with malignant pleural effusion

After we confirmed that the IL-17 concentration was elevated in patients with MPE, we sought possible relationships between IL-17 and gender, age, histologic type of tumor, cancer stage, Eastern Cooperative Oncology Group performance status (ECOG PS), positive cytologic examination and location of pleural effusion. As shown in Table 2, we found no significant correlation between IL-17 concentration and any of these clinicopathological factors.

**Table 2:** **Interleukin 17 levels in pleural effusion of lung cancer patients**^**a**^

**Clinical variables**	**Patients ( *n *)**	**IL-17 (pg/ml) Mean ± SD**	***P*-value**
Age (years)			0.343
≥60	38	27.08 ± 4.87	
<60	40	19.92 ± 5.28	
Gender			0.259
Male	36	21.23 ± 5.30	
Female	42	19.87 ± 5.23	
Histologic type			0.491
Adenocarcinoma	67	20.33 ± 6.12	
Nonadenocarcinoma	11	21.52 ± 5.05	
ECOG PS			0.256
0 or 1	50	19.99 ± 5.37	
2 to 4	28	21.41 ± 5.21	
Cytologic examination			0.912
Positive	60	20.46 ± 5.18	
Negative	18	20.62 ± 6.12	
Stage			0.706
M1a	54	20.34 ± 4.58	
M1b	24	20.83 ± 6.01	
Location			0.751
Right	50	20.34 ± 5.36	
Left	28	20.15 ± 4.97	

^a^ECOG PS, Eastern Cooperative Oncology Group performance status; IL-17, Interleukin 17.

### Prognostic significance of pleural fluid IL-17 for lung cancer patients with malignant pleural effusion

**Figure 3:**
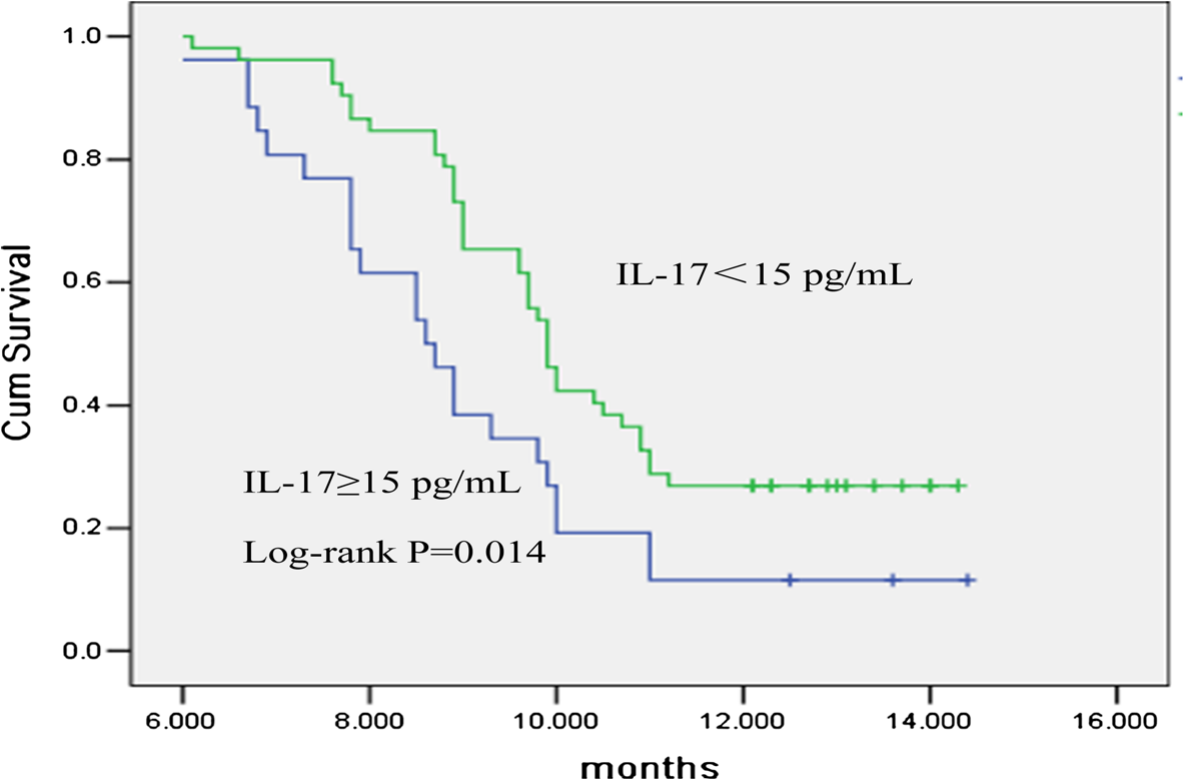
**Relationship between pleural fluid interleukin 17 levels and overall survival in lung cancer patients analyzed by Kaplan–Meier analysis.** The survival of lung cancer patients with pleural fluid interleukin 17 (IL-17) levels <15 pg/ml was significantly longer than the survival of patients with levels ≥15 pg/ml (*P* < 0.05).

The OS for all lung cancer patients in the current study was 6.7 months, and the 1-year survival rate was 21.8%. The prognostic significance of pleural fluid IL-17 concentration and other factors in patients with MPE was evaluated by univariate analysis (Table 3). The cutoff value chosen for pleural fluid IL-17 concentration in lung cancer patients was 15 pg/ml. High pleural fluid IL-17 concentration, older age, late-stage disease and poor ECOG PS were factors associated with poor survival. The survival time in lung cancer patients with pleural fluid IL-17 concentrations below 15 pg/ml was significantly longer than in those with higher concentrations (OS 10.8 vs. 4.7 months; *P* < 0.05) (Figure 3).

**Table 3:** **Univariate analysis for overall survival in lung cancer patients with malignant pleural effusion**^**a**^

**Parameters**	**Patients ( *n *)**	**OS (months)**	***P*-value**
Age (years)			0.038*
≥60	38	5.1	
<60	40	9.4	
Gender			0.538
Male	36	8.7	
Female	42	6.2	
IL-17 (pg/ml)			0.023*
≥15	26	4.7	
<15	52	10.8	
ECOG PS			0.011*
0 or 1	50	9.4	
2 to 4	28	3.1	
Histologic type			0.617
Adenocarcinoma	67	7.5	
Nonadenocarcinoma	11	5.3	
Stage			0.047*
M1a	54	8.6	
M1b	24	4.2	
Location			0.636
Right	50	7.3	
Left	28	6.9	

^a^ECOG PS, Eastern Cooperative Oncology Group performance status; OS, overall survival. *Significant difference.

To test the prognostic value of pleural fluid IL-17 concentration, we performed multivariate analysis of prognostic factors using the Cox proportional hazards model. We found that pleural fluid IL-17 concentration (*P* = 0.007) had independent prognostic significance, whereas ECOG PS (*P* = 0.157), age (*P* = 0.545), tumor location (*P* = 0.362) and cancer stage (*P* = 0.734) lacked significant independent effects on survival (Table 4).

**Table 4:** **Multivariate Cox proportional hazards analysis for overall survival in lung cancer patients with malignant pleural effusion**^**a**^

**Parameters**	**HR**	**95% CI**	***P-*** **value**
Age (years)			0.545
≥60	1		
<60	1.263	0.593 to 2.689	
IL-17 (pg/ml)			0.007*
≥15	1		
<15	0.329	0.146 to 0.742	
ECOG PS			0.157
0 or 1	1		
2 to 4	0.653	0.778 to 4.745	
Location			0.362
Right	1		
Left	0.455	0.083 to 2.479	
Stage			0.734
M1a	1		
M1b	0.168	0.449 to 3.112	

^a^95% CI, 95% confidence interval; ECOG PS, Eastern Cooperative Oncology Group performance status; HR, Hazard ratio; IL-17, Interleukin 17. *Significant difference.

## Discussion

To the best of our knowledge, this study is the first on pleural effusion in which IL-17 levels were investigated for their diagnostic and prognostic power simultaneously in lung cancer patients with MPE. Our findings suggest that profoundly elevated pleural fluid concentrations of IL-17 (≥15 pg/ml) measured at the onset of effusion correlated with shorter survival in lung cancer patients. Furthermore, multivariate analysis of prognostic factors identified pleural fluid IL-17 concentration as an independent prognostic factor for OS. These results suggest that IL-17 concentration measured in pleural effusion is an indicator of not only the presence of lung cancer but also the patient’s survival outcome.

IL-17 is a proinflammatory cytokine produced mainly by CD4^+^ T lymphocytes. It may be important in tumor cell growth and may contribute to the aggressiveness of human tumors [24]. Recently, accumulating evidence has shown that IL-17-positive cells are frequently present in multiple cancers, including prostate cancer [25], colorectal cancer [26], hepatocellular carcinoma [27], breast cancer [28], ovarian cancer [20] and non-small-cell lung cancer [29]. Researchers in some studies have reported that IL-17 cell expression in MPE is elevated [15,23]. Most of the reports indicate that IL-17 may protect against tumors by promoting immune system–mediated tumor rejection [30]. These results suggest the IL-17 plays an important role in tumor immune system evasion. One inference derived from this hypothesis is that IL-17 concentration in pleural effusion could be a good indicator in the follow-up of tumor patients, which is supported by our presently reported results.

Differentiating malignant from nonmalignant pleural effusion is a clinical problem, and conventional methods have proven inadequate [3-6]. A reliable marker for rapid and accurate diagnosis of pleural effusion is greatly needed. In this study, the IL-17 concentrations in malignant pleural effusion were higher than those in nonmalignant and TB pleural effusion. Our results suggest that IL-17 should be a tumor marker for the diagnosis of MPE. No statistically significant correlation was observed between IL-17 concentration and histological type of lung cancer. We assigned 15 pg/ml IL-17 in MPE as the diagnostic cutoff value, which had sensitivity of 79.5% and specificity of 91.1%. This result shows that IL-17 concentration could be a valuable marker in the differential diagnosis of malignant and nonmalignant pleural effusion. Further studies of the potential efficacy of this marker are needed.

## Conclusion

IL-17 concentrations in MPE associated with lung cancer are significantly higher than those in nonmalignant pleural effusion. Determination of IL-17 concentration in pleural effusion is diagnostically informative and IL-17 concentration is an independent prognostic factor that shows promise in the follow-up of lung cancer patients who develop pleural effusion.

## Competing interests

The authors declare that they have no competing interests.

## Authors’ contributions

CHX and PZ collected data and specimens, carried out the ELISA, analyzed the results and drafted the manuscript. CHX and LKY conceived and designed the experiments, drafted and revised the manuscript critically and gave final approval of the version to be published. YZ participated in study coordination and statistical analysis. All authors read and approved the final manuscript.
